# Familial short stature: genetic architecture, risk stratification, and precision management

**DOI:** 10.3389/fendo.2026.1890896

**Published:** 2026-08-25

**Authors:** Chang Zhang, Na Wu, Ming Gong, Min Yang, Lingzhe Meng, Ying Xin

**Affiliations:** Department of Pediatrics, Shengjing Hospital of China Medical University, Shenyang, China

**Keywords:** familial short stature, genetic diagnosis, growth plate disorders, monogenic short stature, precision medicine, recombinant human growth hormone

## Abstract

**Background:**

Familial short stature (FSS) has traditionally been considered a benign growth pattern characterized by short stature clustering within families and has often been regarded as a normal variant of growth. However, recent advances in genomic technologies have demonstrated that a subset of children presenting with an FSS phenotype harbor identifiable monogenic variants, particularly in genes involved in growth plate development and skeletal growth. These findings challenge the traditional phenotype-based understanding of FSS and support an etiology-oriented diagnostic framework.

**Objective:**

To summarize current knowledge regarding the genetic architecture of FSS, review existing clinical risk stratification frameworks for genetic evaluation, and evaluate available evidence regarding treatment outcomes across different genetic etiologies.

**Methods:**

A literature search was performed in PubMed, Embase, and Web of Science from inception to May 2026, using keywords including “familial short stature,” “familial idiopathic short stature,” “genetic testing,” “*ACAN*,” “*SHOX*,” and “*NPR2*”. Relevant original studies and review articles addressing genotype–phenotype correlations, diagnostic yield of genetic testing, or responses to recombinant human growth hormone (rhGH) therapy were considered.

**Results:**

Emerging evidence indicates that monogenic variants can be identified in a subset of children with an FSS phenotype, especially among those with more severe short stature and autosomal dominant inheritance patterns. Variants affecting growth plate biology represent some of the most frequently reported genetic causes of FSS, with *ACAN, SHOX*, and *NPR2* being the most frequently implicated genes. Existing clinical frameworks based on parental height patterns and inheritance characteristics may help stratify patients with FSS according to the likelihood of monogenic etiology and guide selection of individuals who may benefit from genetic testing. Available evidence suggests that rhGH therapy may improve growth outcomes in several monogenic forms of FSS, although treatment responses vary according to genetic etiology.

**Conclusions:**

FSS should be regarded as a heterogeneous clinical phenotype rather than a single diagnostic entity. Integration of existing clinical risk stratification approaches with molecular diagnosis may enable more precise identification of underlying genetic causes and facilitate individualized therapeutic decision-making. Future advances in FSS management will likely depend on precision medicine approaches linking phenotype, genotype, and treatment response.

## 1 Introduction

Familial short stature (FSS) is a clinical descriptive term referring to the recurrent occurrence of short stature within a family after exclusion of overt systemic, endocrine, nutritional, or chromosomal disorders (1–3).

Historically, FSS has been regarded as a normal variant of growth and has long been considered one of the most common explanations for childhood short stature encountered in pediatric endocrine practice (2, 3). Early longitudinal studies demonstrated that many children with FSS followed a growth trajectory consistent with their genetic background, leading to the traditional view of FSS as a benign constitutional growth pattern rather than a pathological condition.

In contemporary pediatric endocrinology, children with a short stature who remain undiagnosed after comprehensive clinical evaluation are classified as having idiopathic short stature (ISS) (4). Among these individuals, those with a positive family history of short stature are often categorized as familial idiopathic short stature (FISS), a subtype formally recognized by the International Classification of Pediatric Endocrine Diagnoses (ICPED) (4). Importantly, FSS and FISS are not synonymous concepts. FSS is a broad phenotypic description based primarily on clinical presentation and family history, whereas FISS is a diagnostic category established only after exclusion of all currently identifiable causes of growth failure (5).

Recent advances in next-generation sequencing have fundamentally altered our understanding of the genetic basis of short stature. Human height is recognized as a highly heritable trait influenced by both common variants with small effects and rare variants with larger phenotypic consequences (6). Increasing evidence indicates that a subset of children presenting with an FSS phenotype may harbor pathogenic monogenic variants, particularly in genes regulating growth plate development, extracellular matrix (ECM) composition, and endocrine signaling pathways (6–9).

Several studies have reported that the diagnostic yield of genetic testing is particularly high among children with familial aggregation of short stature, severe height deficits, or additional subtle skeletal abnormalities (8–10). Consequently, genetic evaluation is increasingly incorporated into the diagnostic assessment of children previously classified as ISS or FSS (10, 11). Beyond establishing a molecular diagnosis, genetic testing may provide information regarding prognosis, treatment responsiveness, recurrence risk, and family counseling (12). However, clear clinical criteria for identifying which children with an FSS phenotype should undergo genetic testing remain incompletely established.

Therefore, the aim of this review is to summarize current evidence regarding the genetic architecture of FSS, review existing clinical risk stratification frameworks for genetic evaluation, and discuss available therapeutic evidence across different genetic subtypes. By integrating phenotype recognition, molecular diagnosis, and genotype-informed treatment considerations, this review provides a clinically oriented perspective on the transition of FSS from a descriptive auxological phenotype toward a genetically informed and clinically meaningful condition.

## 2 Genetic risk stratification of FSS

### 2.1 FSS as a clinical phenotype

FSS should primarily be regarded as a clinical phenotype rather than a specific etiological diagnosis. The presence of short stature in multiple family members may reflect the inheritance of numerous common height-associated variants with small effects, but it may also result from transmission of pathogenic variants with major effects on skeletal growth (6, 8, 13).

Recent genomic studies have demonstrated that a subset of children presenting with an FSS phenotype harbor identifiable monogenic variants (8–10, 13). Importantly, many of these disorders follow autosomal dominant inheritance patterns and therefore produce family histories that closely resemble classical FSS. Consequently, the observation of short stature in multiple generations should not automatically be interpreted as evidence of benign constitutional inheritance.

The recognition of FSS as a heterogeneous phenotype has important clinical implications. Rather than representing a single diagnostic category, FSS may encompass polygenic short stature, monogenic growth plate disorders, mild skeletal dysplasias, and other inherited growth conditions that may be clinically difficult to distinguish during initial evaluation (8, 9).

### 2.2 Clinical risk stratification based on parental height patterns

**Figure 1:**
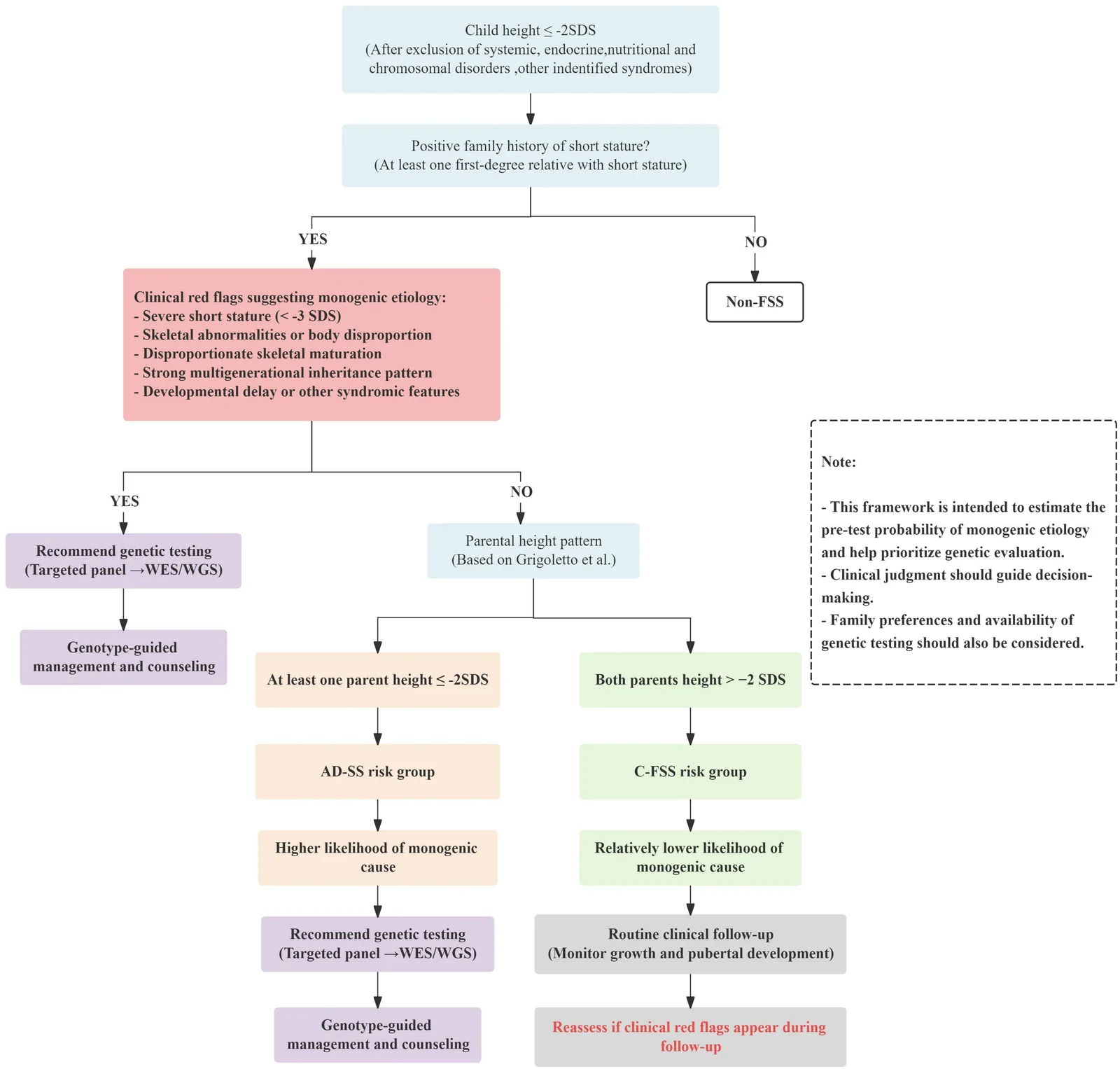
Clinical risk stratification framework for genetic evaluation in familial short stature (adapted from Grigoletto et al.). SDS, standard deviation score; AD-SS, autosomal dominant short stature; C-FSS, constitutional familial short stature; WES, whole-exome sequencing; IWGS, whole-genome sequencing.

To improve identification of patients most likely to benefit from genetic testing, Grigoletto et al. proposed a clinically applicable risk stratification framework that categorizes children with an FSS phenotype into autosomal dominant short stature (AD-SS) and constitutional familial short stature (C-FSS) based on parental height patterns (14). Importantly, this framework was developed as a clinical risk assessment tool rather than a definitive etiological classification. It aims to estimate the likelihood of monogenic disease and guide prioritization of genetic evaluation (**Figure 1**).

AD-SS describes children with height SDS ≤−2.0 and at least one parent with height SDS ≤−2.0, suggesting a potential autosomal dominant inheritance pattern (14). This group is considered at higher risk for monogenic causes and may represent a priority population for genetic evaluation.

In contrast, C-FSS refers to children whose height falls within the expected target height range based on parental stature, while both parents remain within the normal population range (>−2 SDS) (14). In these cases, short stature is more likely to reflect polygenic inheritance rather than a highly penetrant monogenic disorder. Although children classified as C-FSS are generally considered less likely to harbor highly penetrant monogenic disorders, this classification does not exclude the possibility of an underlying genetic defect. Assortative mating between individuals with short stature, variable penetrance, and the coexistence of polygenic and monogenic determinants may result in families in which both parents are short despite the presence of an autosomal dominant pathogenic variant. Therefore, genetic testing may still be appropriate in selected C-FSS patients, particularly when severe short stature, disproportion, abnormal bone age patterns, skeletal abnormalities, or a strong multigenerational family history is present.

Although this classification does not establish a definitive diagnosis, it provides a practical framework for prioritizing genetic investigations in clinical practice and may improve the efficiency of genetic evaluation strategies (14).

### 2.3 Clinical indicators suggesting monogenic etiology

Not all children with an FSS phenotype have the same likelihood of harboring monogenic variants. Several clinical features have been associated with a higher likelihood of identifying pathogenic variants.

The severity of height deficit represents an important predictor of monogenic disease. Studies have shown that the prevalence of pathogenic variants increases as stature becomes progressively shorter, particularly among children with height SDS below −3.0 (8, 15–18).

Family history may also provide important clues. The presence of multiple affected individuals across successive generations is suggestive of autosomal dominant inheritance and should raise suspicion for monogenic growth disorders (8, 10).

In addition, specific auxological and radiological findings may help identify children who warrant genetic evaluation. Disproportionate body segments, mesomelia, Madelung deformity, abnormal bone age patterns, brachydactyly, or subtle skeletal abnormalities have all been associated with pathogenic variants in genes such as *SHOX, ACAN*, and *NPR2* (16, 19–21). Even when these abnormalities are mild, their presence may substantially increase the likelihood of establishing a molecular diagnosis.

Collectively, these observations support a phenotype-guided approach in which genetic testing is prioritized for children with severe short stature, dominant familial inheritance patterns, or additional clinical indicators suggestive of specific genetic disorders (17, 18).

### 2.4 Clinical implications of risk stratification

The primary goal of risk stratification is not merely to identify genetic variants but to improve clinical management. Establishing a molecular diagnosis may provide information regarding disease mechanisms, expected growth trajectories, treatment responsiveness, and recurrence risk within affected families (12).

Increasing evidence suggests that molecular diagnosis may also influence therapeutic decision-making. For example, children with variants in *ACAN*, *SHOX*, or *NPR2* may show variable but potentially favorable responses to recombinant human growth hormone (rhGH) therapy, whereas treatment outcomes may differ substantially across other genetic etiologies (10, 12, 22–24). Identification of the underlying genetic cause therefore has potential implications for patient selection, treatment timing, and long-term follow-up.

Ultimately, integrating clinical phenotyping with molecular testing represents an important step toward precision medicine in pediatric growth disorders. Such an approach allows clinicians to move beyond descriptive classifications and establish management strategies tailored to the biological basis of individual growth impairment (9, 12, 25).

## 3 Genetic architecture of FSS

### 3.1 Polygenic and monogenic contributions to FSS

Human height is one of the most heritable quantitative traits, with genetic factors accounting for approximately 80% of the observed variation in adult stature (6). Recent large-scale genomic studies have demonstrated that height determination exists on a continuous spectrum, ranging from the cumulative effects of thousands of common variants with small individual effects to rare pathogenic variants with large phenotypic consequences (26).

Traditionally, FSS has been regarded as a polygenic trait resulting from the inheritance of multiple height-lowering alleles across generations (13). Genome-wide association studies (GWAS) have identified thousands of loci associated with human height, supporting the concept that many individuals with mild FSS represent the lower end of the normal distribution of polygenic growth potential (6, 13, 26).

However, the rapid implementation of next-generation sequencing has fundamentally altered this view. Increasing evidence indicates that a considerable proportion of selected children with an FSS phenotype may harbor identifiable monogenic variants. In a landmark cohort study, Plachy et al. reported that pathogenic monogenic variants were detected in approximately 38% of children with FSS, particularly among those with more severe height deficits and autosomal dominant inheritance patterns (8). These findings suggest that FSS should no longer be viewed as a purely polygenic condition but rather as a heterogeneous clinical entity encompassing both polygenic and monogenic growth disorders (9).

Current evidence indicates that genetic causes of FSS involve several biological pathways, including growth plate development, GH–IGF-1 signaling, intracellular signaling cascades, and ECM organization (9, 26). Understanding these mechanisms provides the foundation for genetic diagnosis and precision management (**Table 1**).

**Table 1:** Monogenic causes of familial short stature and their characteristic phenotypes.

Pathway	Gene	Typical phenotype	Reported cases	rhGH-treated cases	Reference
Growth plate genes	ACAN	• Advanced bone age (variable, may be absent or only mildly advanced in some patients) • Mild brachydactyly • Early growth cessation	91	61	(8, 10, 15, 20, 21, 23, 27–38, 73–76)
	SHOX	• Increased sitting-height/height ratio • Madelung deformity or subtle wrist changes • Short limbs or mesomelic disproportion	23	5	(8, 19, 39, 40, 81–85)
	NPR2	• Mild skeletal abnormalities(subtle hand X-ray changes) • Occasionally disproportionate body segments • No consistent bone age advance	39	32	(8, 22, 24, 44, 72, 86–89)
	FGFR3	• Mild skeletal dysplasia features • disproportionate short stature • subtle radiographic abnormalities	6	0	(8, 10, 46, 47, 99)
GH–IGF1 axis genes	GHSR	• Moderate obesity (elevated BMI) • Relatively low GH peak • Possible family history of short stature with obesity	5	3	(8, 10, 48, 51)
	IGF1R	• Intrauterine growth restriction • Persistent postnatal growth impairment	1	0	(10, 48, 54–56)
	GH1	• Isolated growth hormone deficiency • Proportionate short stature	/	/	(48, 49)
	GHRHR	• Isolated growth hormone deficiency; • Proportionate short stature • Autosomal recessive inheritance	/	/	(48, 49)
	GHR	• GH insensitivity • Low IGF-1 • Severe postnatal growth failure	/	/	(48, 50)
	STAT5B	• GH insensitivity; • Variable immune abnormalities	/	/	(48, 52, 53)
	IGF1	• Primary IGF-1 deficiency • Severe prenatal and postnatal growth	/	/	(48, 56)
RAS/MAPK pathway genes	PTPN11	• Subtle facial features (hypertelorism, low-set ears) • Possible congenital heart disease	3	0	(8–10, 57)
	NF1	• Skin abnormalities • Mild learning difficulties	2	0	(8–10, 57)
	SOS1	• Noonan-like phenotype	2	0	(8–10, 57)
	RAF1	• Noonan-like phenotype	/	/	(9, 57)
	KRAS	• Noonan-like phenotype	/	/	(9, 57)
	SHOC2	• Noonan-like phenotype	/	/	(9, 57)
ECM and collagen genes	COL2A1	• Mild spondyloepiphyseal dysplasia • Joint laxity or early osteoarthritis • Possible myopia	14	5	(8, 10, 58–60)
	COL11A1	• Skeletal abnormalities • Auditory system abnormalities	10	4	(8, 10, 58–60)
	COL11A2	• Bone fragility or blue sclera • Dentinogenesis imperfecta (mild)	4	3	(8, 58–60)
	COL9A1	• Multiple epiphyseal dysplasia • Early-onset osteoarthritis	/	/	(58, 61, 63, 64)
	COL9A2	• Multiple epiphyseal dysplasia; • Early-onset osteoarthritis	/	/	(58, 61, 63, 64)
	COL9A3	• Multiple epiphyseal dysplasia • Early-onset osteoarthritis	/	/	(58, 61, 63, 64)
	COL10A1	• Schmid metaphyseal chondrodysplasia • Bowed legs • Waddling gait	/	/	(58, 62, 64)
Other emerging genes	TRHR	• Insufficient data	2	0	(8, 10)
	MBTPS2	• Insufficient data	2	0	(8, 10)
	IGFALS	• Insufficient data	2	0	(8, 10)
	HMGA2	• Insufficient data	2	0	(8, 10)
	OTX2	• Insufficient data	1	0	(8)
	MATN3	• Insufficient data	1	0	(8)
	FLNB	• Insufficient data	1	0	(10)
	SALL4	• Insufficient data	1	0	(8)
Total	**/**	**/**	**212**	**113**	**/**

“/” denotes the absence of reported FSS-specific cases.Bold values indicate the total number of reported cases and rhGH-treated cases among the listed monogenic causes of familial short stature.

### 3.2 Growth plate genes: the major contributors of monogenic FSS

Growth plate dysfunction has emerged as the predominant mechanism underlying monogenic FSS (9, 10). The growth plate is a highly organized cartilage structure responsible for longitudinal bone growth, and disruption of chondrocyte proliferation, differentiation, or ECM formation may result in impaired linear growth.

Among currently identified monogenic causes, pathogenic variants in the *ACAN* gene represent the most frequently reported genetic defect associated with FSS (8, 10). *ACAN* encodes aggrecan, a major proteoglycan component of the ECM within growth plate cartilage. Aggrecan provides structural support and hydration to cartilage tissue and is essential for normal endochondral ossification (20, 27–31). Several studies have demonstrated that *ACAN* variants are enriched in FSS cohorts. Furthermore, the prevalence of *ACAN* variants increases with increasing severity of parental short stature, suggesting a strong genotype–phenotype correlation (15, 32, 33). Advanced bone age is traditionally considered a characteristic feature of *ACAN*-related short stature (21). Nevertheless, subsequent studies have demonstrated considerable phenotypic variability, with some patients exhibiting normal or even delayed bone age (30, 34). Therefore, the absence of advanced bone age should not exclude *ACAN* testing when clinical suspicion is high. Expanding evidence from large cohort studies has further characterized the phenotypic spectrum of *ACAN*-related short stature, demonstrating considerable variability in bone age patterns, skeletal manifestations, and treatment responses (35–38).

The *SHOX* gene is located within the pseudoautosomal region of the X and Y chromosomes and plays a critical role in growth plate development (19). *SHOX* functions as a transcription factor regulating chondrocyte proliferation and differentiation. Haploinsufficiency of *SHOX* leads to a broad phenotypic spectrum ranging from isolated short stature to Leri–Weill dyschondrosteosis and Langer mesomelic dysplasia (19, 39, 40). Body disproportion remains one of the most important clinical clues for identifying *SHOX* deficiency. Increased sitting-height-to-height ratio and abnormal extremity-to-trunk ratio have been proposed as useful screening markers in children with FSS (41–43).

The *NPR2* gene encodes natriuretic peptide receptor-B (NPR-B), the primary receptor for C-type natriuretic peptide (CNP) (44). Activation of the CNP/NPR-B signaling pathway stimulates cyclic guanosine monophosphate (cGMP) production and promotes chondrocyte proliferation while antagonizing *FGFR3*-mediated growth inhibition (44). Recent studies have demonstrated that heterozygous *NPR2* variants are relatively common among children with FSS, with reported detection rates ranging from 5.7% to 13.6% (22, 44). Long-term observational studies further suggest that *NPR2*-related short stature exhibits substantial phenotypic heterogeneity but generally favorable responses to growth hormone treatment (24). Functional studies have suggested that *NPR2* variants may influence chondrocyte differentiation through additional signaling pathways, including JAK2-STAT5 signaling (45).

The *FGFR3* gene encodes fibroblast growth factor receptor 3, a key negative regulator of growth plate activity. Activation of *FGFR3* signaling inhibits chondrocyte proliferation and differentiation through downstream pathways such as MAPK signaling, thereby restricting endochondral bone growth. Pathogenic *FGFR3* variants are classically associated with skeletal dysplasias, including achondroplasia and hypochondroplasia (46). However, milder variants may occasionally present with relatively isolated short stature and mimic an FSS phenotype (47). Therefore, *FGFR3*-related disorders should be considered in children with disproportionate short stature, subtle skeletal abnormalities, or atypical features suggestive of growth plate dysfunction.

Collectively, *ACAN, SHOX*, and *NPR2*, and other growth plate-related genes such as *FGFR3* represent important genetic contributors to monogenic short stature, although their phenotypic spectrum extends beyond classic FSS.

### 3.3 GH–IGF-1 axis-related genes

The GH–IGF-1 axis is a critical endocrine pathway regulating childhood growth. Although growth plate defects represent the predominant genetic mechanism underlying monogenic FSS, variants affecting GH secretion, receptor signaling, or IGF-1 action may occasionally present with familial clustering of short stature and overlap phenotypically with FSS and should be considered during genetic evaluation (48).

Genetic abnormalities involving the GH–IGF-1 axis can impair growth through defects in hormone production, receptor activation, or downstream signaling. Defects affecting GH secretion include pathogenic variants in *GH1, GHRHR*, and *GHSR*, which may impair GH synthesis, hypothalamic regulation, or ghrelin-mediated stimulation of GH release, respectively, resulting in reduced activation of the GH–IGF-1 axis (48–51). Although these disorders are generally classified as isolated growth hormone deficiency (IGHD) rather than FSS, milder forms may present with an FSS phenotype and are clinically relevant because affected individuals may benefit from rhGH therapy.

Abnormalities in GH signaling and IGF-1 action represent additional mechanisms of growth impairment. Pathogenic variants in *GHR*, encoding the growth hormone receptor, cause GH insensitivity syndrome through impaired GH signaling and reduced IGF-1 production (50). Similarly, defects in downstream signaling components such as *STAT5B* may result in impaired GH signaling, short stature, and variable immune abnormalities (52, 53). Variants affecting *IGF1* or *IGF1R* may directly impair IGF-1 production or cellular responsiveness to IGF-1, respectively, leading to growth impairment in affected individuals (54–56).

Although GH–IGF-1 axis-related disorders are less frequently identified than growth plate defects in FSS cohorts, their recognition remains clinically important because molecular diagnosis may influence endocrine evaluation, treatment selection, and interpretation of response to rhGH therapy.

### 3.4 RAS/MAPK signaling pathway genes

The RAS/MAPK signaling pathway is a highly conserved intracellular cascade that regulates cell proliferation, differentiation, and developmental processes. Abnormal activation or dysregulation of this pathway can affect skeletal growth and contribute to short stature, particularly in individuals with RASopathies (9, 57).

Pathogenic variants in genes involved in the RAS/MAPK pathway, including *PTPN11*, *SOS1*, *RAF1*, *KRAS*, and *SHOC2*, are primarily associated with Noonan syndrome and related disorders (9, 57). Although many affected individuals exhibit characteristic syndromic features, some individuals may have mild or atypical presentations dominated by growth impairment, potentially leading to misclassification as FSS.

Among these genes, *PTPN11* is one of the most frequently implicated genes in Noonan syndrome and plays an important role in regulating intracellular growth signaling. Other pathway genes, including *SHOC2* and *KRAS*, have been associated with variable phenotypic presentations ranging from typical RASopathies to milder growth abnormalities (57).

Therefore, children with an FSS phenotype accompanied by dysmorphic features, developmental abnormalities, cardiac manifestations, or other syndromic clues should undergo careful clinical reassessment and consideration of molecular testing. Identification of RAS/MAPK pathway variants not only clarifies diagnosis but also provides important information regarding prognosis, surveillance, and genetic counseling.

### 3.5 ECM and collagen genes

The ECM provides structural support for growth plate cartilage and is essential for normal endochondral ossification. Genetic defects affecting ECM components may disrupt cartilage organization and longitudinal bone growth, resulting in a broad spectrum of skeletal phenotypes ranging from mild short stature to recognizable skeletal dysplasias (58).

Several collagen genes involved in cartilage development have been associated with short stature phenotypes that may overlap with FSS. Variants in *COL2A1*, *COL11A1*, and *COL11A2* are well-established causes of type II collagenopathies and can present with variable degrees of skeletal involvement, including disproportionate or proportionate short stature (58–60). In addition, defects in other cartilage-related collagen genes, including *COL9A1*, *COL9A2*, *COL9A3*, and *COL10A1*, have been associated with growth plate abnormalities and skeletal dysplasia phenotypes that may overlap with short stature presentations (61–64). However, these conditions usually belong to the skeletal dysplasia spectrum rather than representing classic FISS. Careful clinical evaluation for subtle skeletal features is therefore required.

Therefore, ECM and skeletal development-related genes represent an important but less common category of genetic causes in children presenting with an FSS phenotype. Recognition of these disorders is particularly relevant in patients with disproportion, abnormal radiographic findings, advanced bone age, or other subtle skeletal manifestations, in whom genetic evaluation may clarify diagnosis and guide clinical management.

### 3.6 From genetic architecture to precision diagnosis

The expanding understanding of FSS genetics has shifted the diagnostic paradigm from a phenotype-based classification toward a molecularly informed approach. FSS should be considered a genetically heterogeneous phenotype encompassing both polygenic growth variation and monogenic growth disorders (9, 26).

Genetic testing should be prioritized in children with clinical features suggestive of monogenic disease, including severe short stature, autosomal dominant inheritance, body disproportion, abnormal bone age, skeletal abnormalities, or additional syndromic features (8–10, 16). Identification of pathogenic variants can refine diagnosis, improve genetic counseling, provide prognostic information, and influence therapeutic decision-making.

The increasing availability of next-generation sequencing has further expanded the feasibility of molecular diagnosis. Recent studies have demonstrated meaningful diagnostic yields of exome sequencing in children with short stature, particularly among those with additional clinical abnormalities or unexplained growth impairment (65, 66).

Therefore, integrating auxological characteristics, family history, and genomic information represents an essential step toward precision diagnosis in FSS. Future strategies should focus on developing risk stratification models to identify individuals most likely to benefit from genetic testing and individualized growth management (9, 16, 26).

## 4 Treatment and future therapeutic perspectives

### 4.1 Principles of rhGH therapy in FSS

At present, no specific international guidelines exist for the management of FSS. In clinical practice, therapeutic decisions are largely extrapolated from recommendations for ISS, particularly when patients fulfill regulatory criteria for rhGH treatment.

Historically, FSS was considered a benign growth variant with limited therapeutic implications. However, increasing recognition of monogenic growth disorders within the FSS spectrum has demonstrated substantial heterogeneity in treatment responsiveness.

The response to rhGH therapy is influenced by multiple factors, including age at treatment initiation, baseline height SDS, pubertal status, treatment duration, bone age, and underlying genetic etiology. Earlier initiation and longer treatment duration are generally associated with greater height gain across different causes of short stature, reflecting the preservation of residual growth potential rather than an *ACAN*-specific phenomenon.

Therefore, genetic diagnosis may provide additional information beyond confirming etiology, helping clinicians predict treatment responsiveness, optimize timing of intervention, and establish individualized management strategies (12).

### 4.2 Genotype-specific responses to rhGH therapy

#### 4.2.1 *ACAN*-related FSS

*ACAN*-related FSS represents the monogenic FSS subtype with the largest body of clinical evidence regarding rhGH treatment response (10, 23).

Published studies have reported improvements in height SDS ranging from +0.1 to +1.9 after rhGH treatment (10, 15, 23, 28, 31, 33, 67–76). Treatment response appears to be influenced by several factors. In *ACAN*-related patients, however, treatment timing may be particularly critical because accelerated skeletal maturation may reduce the duration of effective growth-promoting therapy.

Our exploratory analysis of published individual-level data suggested a numerical difference in short-term height SDS improvement according to skeletal maturation status (**Table 2**), with higher values observed in patients with advanced bone age. However, this finding should not be interpreted as evidence that advanced bone age predicts better rhGH responsiveness, given the limited sample size and substantial heterogeneity in treatment duration, age at initiation, and genetic background.

**Table 2:** Summary of rhGH treatment outcomes in major monogenic forms of familial short stature.

Gene	N (with outcome)	Age at GH initiation (years), median (IQR)	Baseline height SDS, median (IQR)	ΔHeight SDS, median (IQR)	Key observations
**ACAN**	61	6.4(4.1–9.5)	–3.2(–3.8 to–2.7)	+0.6(+0.2 to+1.0)	Better outcomes with earlier treatment; advanced bone age may limit response
**SHOX**	5	6.0(4.0–7.5)	−3.2(−3.6 to−2.8)	+0.7(+0.4 to+0.9)	Approved indication; consistent growth improvement
**NPR2**	32	7.5(4.6–11.7)	−3.1(−3.7 to−2.6)	+0.9(+0.4 to+1.6)	Generally favorable response; possible genotype-specific differences

N indicates the number of patients with available pre- and post-treatment height SDS data used to calculate median ΔSDS.

For ACAN, 61 patients received rhGH in the literature; for NPR2, 23 patients received rhGH. Data were extracted from published cases (see **Supplementary Tables S1**-**S4** for individual patient data).

Unlike many other genetic causes of short stature, *ACAN*-related FSS is often, although not invariably, associated with advanced skeletal maturation, which represents an important therapeutic challenge by reducing the remaining period of linear growth (77). However, skeletal maturation abnormalities are variable, and some individuals may present with normal or only mildly advanced bone age, reflecting the intrinsic growth plate dysfunction caused by aggrecan deficiency rather than accelerated skeletal maturation alone.

Because advanced bone age may accelerate epiphyseal fusion, adjunctive approaches aimed at delaying skeletal maturation have been considered in selected *ACAN*-related patients. Gonadotropin-releasing hormone analogs (GnRHa) have been explored as an adjunctive approach to suppress pubertal progression and potentially prolong the growth period, although current evidence remains limited and is primarily based on small observational studies (78).

In addition, aromatase inhibitors (AIs), including anastrozole and letrozole, have been investigated as adjunctive therapies with rhGH to delay epiphyseal maturation and prolong the period of linear growth, particularly in pubertal boys with limited growth potential (79). However, evidence supporting AI use specifically in *ACAN*-related short stature is currently lacking, and available data mainly derive from boys with idiopathic short stature or GH deficiency (79, 80).

Recent real-world data further confirm the effectiveness of rhGH in *ACAN*-deficient children and support early molecular diagnosis to maximize treatment opportunities (23).

#### 4.2.2 *SHOX*-related FSS

*SHOX* deficiency is currently the only monogenic form of FSS for which rhGH therapy has received regulatory approval. The FDA approved rhGH treatment for *SHOX* deficiency in 2006 based on randomized clinical trials demonstrating significant improvements in height velocity and adult height outcomes (81, 82). Although *SHOX* defects account for approximately 2%–15% of children with short stature in unselected ISS cohorts (19), the prevalence in strictly defined FSS cohorts may be lower or remains to be fully determined, and only a limited number of studies have specifically reported treatment outcomes in patients meeting strict criteria for FSS (19, 40).

Available evidence suggests that rhGH treatment is highly effective in *SHOX*-related FSS, with reported gains in height SDS ranging from +0.4 to +1.94 (40, 83–85). Interestingly, genotype may influence treatment response. Preliminary observations indicate that patients carrying coding-region variants may exhibit greater growth responses than those with enhancer-region deletions, although larger studies are needed to confirm this finding.

Overall, *SHOX* deficiency currently represents one of the strongest examples of successful genotype-guided therapy in pediatric growth disorders.

#### 4.2.3 *NPR2*-related FSS

The *NPR2* gene encodes natriuretic peptide receptor-B (NPR-B), a key component of the CNP signaling pathway involved in growth plate regulation. Heterozygous *NPR2* variants represent one of the most frequently identified monogenic causes of FSS (22, 24).

To date, approximately 80% of reported *NPR2*-related FSS patients have received rhGH therapy (24). Across published cohorts, the mean improvement in height SDS is approximately +1.0, indicating a generally favorable therapeutic response (8, 22, 24, 44, 72, 86–89).

Interestingly, genotype-specific differences may exist. Recent observations suggest that patients carrying truncating variants may achieve greater height gains than those with missense variants, although this finding requires confirmation in larger cohorts (89).

The multicenter real-world study by Renes et al. confirmed sustained growth acceleration during long-term rhGH therapy in children with *NPR2* haploinsufficiency, supporting rhGH as an effective treatment option for this subgroup (24).

Taken together, current evidence indicates that *ACAN*-, *SHOX*-, and *NPR2*-related FSS represent the monogenic FSS subtypes with the strongest evidence supporting rhGH efficacy (**Table 3**). However, the majority of available evidence remains derived from observational studies, and genotype-specific treatment algorithms have not yet been established.

**Table 3:** Exploratory comparison of rhGH treatment outcomes according to skeletal maturation status in children with ACAN-related short stature.

Skeletal maturation category	N	Age at GH initiation (y) median(IQR)	ΔHeight SDS		1st-year ΔHeight SDS		ΔHeight SDS/year	
			Median(IQR)	Range	Median(IQR)	Range	Median(IQR)	Range
Advanced bone age	26	6.45 (4.35-11.6)	0.55 (0.23-0.82)	−0.71 to 1.90	0.80 (0.40-0.80)	0.22 to 1.80	0.53(0.26–0.90)	0.09 to 1.74
Normal or delayed bone age	17	7.9 (6-11.8)	0.40 (0.08-0.80)	−0.30 to 1.85	0.25 (0.03-0.60)	-0.03 to 1.30	0.38 (0.02–0.60)	0 to 1.85

N denotes the number of patients with available data for the corresponding outcome. ΔHeight SDS represents the change in height SDS from baseline to the last follow-up. First-year growth response data were available from a limited number of studies (advanced bone age: n=5; normal/delayed bone age: n=7). Annualized ΔHeight SDS was calculated among patients treated for ≥1 year (advanced bone age: n=11; normal/delayed bone age: n=11). No formal statistical comparison was performed due to heterogeneity among published studies and limited sample size. Individual patient-level data are provided in **Supplementary Table S2**.

### 4.3 Emerging precision therapies

The expanding understanding of the molecular mechanisms underlying FSS has stimulated interest in targeted therapies that directly address disease-specific pathways rather than relying solely on growth hormone stimulation.

#### 4.3.1 CNP analogs

The CNP pathway has emerged as one of the most promising therapeutic targets in skeletal growth disorders.

Activation of NPR-B signaling stimulates chondrocyte proliferation and ECM synthesis while antagonizing *FGFR3*-mediated growth inhibition. Because *NPR2* and *FGFR3* play critical roles in several forms of monogenic short stature, pharmacological enhancement of CNP signaling has attracted considerable attention (9, 90).

Vosoritide, a long-acting CNP analog, has demonstrated efficacy in promoting linear growth in children with achondroplasia in randomized clinical trials (91). Long-term extension studies have further shown sustained increases in growth velocity with continued treatment and with continued monitoring of safety outcomes (92, 93).

Although currently approved only for achondroplasia, its mechanism of action suggests potential applicability to selected forms of FSS involving abnormalities of the *NPR2*/CNP/*FGFR3* axis. Future clinical studies are needed to evaluate its role in genetically defined FSS populations. Emerging evidence also suggests that CNP can mediate skeletal growth even in the absence of growth hormone, raising the possibility that CNP analogs might benefit selected patients with FSS who do not respond optimally to rhGH (94).

#### 4.3.2 RNA-based therapeutic strategies

Recent advances in RNA therapeutics and extracellular vesicle-based delivery systems have opened new possibilities for mechanism-based treatment strategies (95).

In a landmark preclinical study, Yuan et al. developed engineered exosomes capable of delivering growth plate-targeted siRNA and growth hormone molecules. In animal models of ISS, silencing of the long non-coding RNA ISSRL significantly enhanced growth plate activity and promoted longitudinal bone growth (96).

Although these approaches remain experimental, they represent an important proof of concept for future precision therapies capable of correcting disease mechanisms at the molecular level.

#### 4.3.3 Gene-based therapies

Rapid advances in genome editing technologies, including CRISPR-Cas systems and gene replacement approaches, have generated interest in potential future therapies for monogenic growth disorders (97, 98).

Although clinical application remains distant, disorders caused by haploinsufficiency of genes such as *ACAN*, *SHOX*, and *NPR2* may theoretically become candidates for future gene-targeted approaches. Continued progress in gene-editing safety and delivery systems will be essential before such approaches can enter clinical practice.

### 4.4 Future perspectives for precision management

The management of FSS is undergoing a transition from phenotype-based treatment toward genotype-guided precision medicine.

Historically, treatment decisions were based primarily on auxological criteria, including height SDS, growth velocity, and predicted adult height. However, accumulating evidence demonstrates that identical phenotypes may arise from markedly different molecular mechanisms, each associated with distinct prognostic and therapeutic implications.

Future clinical management is therefore likely to involve three integrated components: early clinical risk stratification, identifying patients at increased likelihood of monogenic disease; comprehensive genomic testing, enabling precise molecular diagnosis; mechanism-based therapy, including rhGH, CNP analogs, RNA therapeutics, and future gene-targeted interventions.

Under this framework, the traditional concept of FSS as a benign familial growth variant is being replaced by a biologically informed model linking phenotype, genotype, and treatment response (25). Such an approach has the potential to improve diagnostic accuracy, optimize therapeutic outcomes, and ultimately establish precision growth medicine as the standard of care for children with FSS.

## 5 Limitations and future directions

Despite the significant progress summarized in this review, several limitations should be acknowledged. First, most of the included studies originated from tertiary referral centers, which may introduce selection bias and overestimate the true prevalence of monogenic variants in the general FSS population. Second, the majority of evidence on treatment responses comes from observational studies or small cohorts; large, prospective randomized controlled trials comparing rhGH responses across different genetic etiologies are still lacking. Third, the clinical utility of polygenic risk scores (PRS) in differentiating benign C-FSS from monogenic AD-SS remains unexplored. Fourth, data on non-European populations are limited, and the generalizability of current findings to diverse ethnic groups requires further validation.

Looking forward, several key questions warrant investigation. (1) Can PRS be integrated with clinical parameters to improve the pretest probability of finding a monogenic variant? (2) What are the long-term safety and efficacy profiles of CNP analogs and RNA-based therapies in children with FSS, particularly regarding growth plate-specific delivery and off-target effects? (3) How should the clinical definition of FSS be updated to incorporate molecular findings, and what are the implications for diagnostic coding and clinical management? Addressing these questions will be essential for translating current genetic knowledge into tangible improvements in patient care.

## 6 Conclusion

FSS should no longer be considered a single clinical entity or merely a benign familial growth variant. Emerging evidence demonstrates that FSS represents a biologically heterogeneous spectrum encompassing both polygenic inheritance and a substantial proportion of monogenic growth disorders.

Recent advances in genomic medicine have fundamentally reshaped the understanding of FSS by revealing the important contribution of pathogenic variants in growth plate genes, GH–IGF-1 axis genes, RAS/MAPK signaling genes, and extracellular matrix-related genes. Among these, defects involving *ACAN*, *SHOX*, and *NPR2* appear to account for a significant proportion of genetically diagnosed cases.

The existing clinical risk stratification framework provides a practical approach for identifying patients most likely to benefit from genetic testing. Furthermore, accumulating evidence indicates that treatment response varies according to genetic etiology, highlighting the importance of integrating molecular diagnosis into therapeutic decision-making.

As precision medicine continues to evolve, future management of FSS will increasingly depend on the combination of clinical phenotyping, genomic testing, and mechanism-based therapies. This transition from phenotype-based classification to genotype-guided management has the potential to improve diagnostic accuracy, optimize therapeutic outcomes, and establish a new paradigm for the care of children with FSS.
