# Supplementary material for: Familial short stature: genetic architecture, risk stratification, and precision management
Source: Front Endocrinol (Lausanne). 2026 Aug 25;17:1890896. doi: 10.3389/fendo.2026.1890896 (PMC13551065; doi:10.3389/fendo.2026.1890896)
Supplement: Supplementary file 1 [file Table1.docx]

**Supplementary Tables S1.Clinical characteristics of patients with ACAN gene variant**

| ACAN Variant | Sex | Age at measurement(y) | BA-CA  GH(y) | Birth weight  (kg)(SDS) | | Birth height(cm)(SDS) | | Height  (cm)(SDS) | | Weight(kg) | BMI(kg/m^2^) | Peak GH  (ng/ml) | IGF-1  (ng/ml) | IGFBP3  (μg/ml) | Reference |
| --- | --- | --- | --- | --- | --- | --- | --- | --- | --- | --- | --- | --- | --- | --- | --- |
| c.4852C>T(p.Gln 1618*） | M | 11.5 | -0.2 | 3.0 | 0.00 | 45 | −1.36 | 119.2 | −4.0 | NA | NA | 9.3 | Normal | Normal | [22] |
|  |  | 12.5 | -1 | 3.0 | 0.00 | 45 | −1.36 | 124.9 | -3.8 | NA | NA | NA | NA | NA |  |
| c.4852C>T(p.Gln 1618*） | M | NA | NA | NA | NA | NA | NA | NA | -3.1 | NA | NA | NA | NA | NA | [22] |
| c.7465 T>C(p.Gln2364Pro) | F | 5.6 | 1 | 3 | NA | 50 | NA | 104.5 | -2.09 | 18 | 16.48 | 12.1 | 290 | 4.15 | [24] |
| c.7465 T>C(p.Gln2364Pro) | M | 6.4 | -0.4 | 3.5 | NA | 50 | NA | 100.5 | −3.74 | 17.5 | 17.33 | 15.1 | 148 | 3.43 | [24] |
| t(10;15)(q22.3;q26.1) | F | 59.8 | NA | NA | NA | NA | NA | 139.7 | –3.56 | NA | NA | NA | NA | NA | [23] |
| t(10;15)(q22.3;q26.1) | F | 54.9 | NA | NA | NA | NA | NA | 136 | –4.18 | NA | NA | NA | NA | NA | [23] |
| t(10;15)(q22.3;q26.1) | F | 11.9 | 0.0 | 2.7 | -0.45 | 44 | −1.7 | 128.7 | −2.80 | NA | NA | NA | NA | NA | [23] |
| t(10;15)(q22.3;q26.1) | F | 5.7 | 0.0 | 3.1 | -0.74 | 47.2 | −1.6 | 103.1 | −1.90 | 16.8 | 15.80 | NA | NA | NA | [23] |
| c.6193delC（p.Gln2065 Serfs*27） | M | 9.8 | 0.0 | 3.5 | NA | 48 | NA | 115 | -4 | 25.4 | 19.21 | 5.89 | 308 | 4.44 | [70] |
| c.6193delC（p.Gln2065 Serfs*27） | M | 7 | 0.8 | 3.2 | NA | 50 | NA | 107 | -4 | 22.6 | 19.74 | 7.35 | 189 | 3.98 | [70] |
| c.5026_5027 del（p.Ser 1676 Ter） | M | 4.1 | 1.4 | 2.9 | NA | 50 | NA | 90.5 | -3.6 | 13.5 | 14.96 | 20.4 | 67.5 | 3.1 | [29] |
| c.5026_5027 del(p.Ser 1676 Ter) | F | 9.5 | 2.0 | 3.0 | NA | NA | NA | 122.2 | -2.5 | 29.5 | 19.76 | 24.6 | 135 | 3.02 | [29] |
| c.1504C>T(p.R 502 C) | M | 3.0 | -1.5 | 2.9 | NA | NA | NA | 82 | -3.9 | 12 | 17.85 | 3.91 | 80.7 | 3.2 | [29] |
| c.1411C>T(p.Gln471*) | F | 4.7 | 2.3 | 3.02 | NA | 45 | NA | 92.8 | -3.48 | 13 | 15.10 | NA | 119 | NA | [25] |
| c.1817C>A(p.Ala606Asp) | M | 9.4 | -1.4 | 2.8 | NA | 48 | NA | 120.6 | -2.88 | 22 | 15.13 | 4.63 | 17.4 | NA | [25] |
| c.1762C>T(p.Gln588*) | M | 9.8 | -2.3 | 2.8 | NA | 50 | NA | 114.3 | -4.18 | 22.5 | 17.22 | NA | 111 | NA | [25] |
| c.2266G>C(p.Gly756Arg) | M | 3.3 | 0.7 | 3.5 | NA | 50 | NA | 89.2 | -2.92 | 15.0 | 18.85 | 5.06 | 138 | NA | [25] |
| c.7469G>A(p.Cys2490Tyr) | M | 14.6 | -2.6 | 2.9 | NA | 49 | NA | 137.3 | -4.53 | 44.5 | 23.61 | 8.13 | 399 | NA | [25] |
| c.1733-1G>A | F | 20.4 | Closure | 2.95 | NA | NA | NA | 143.2 | -3.24 | 48 | 23.41 | NA | NA | NA | [25] |
| c.711051del(p.His25Thr350del) | F | 4.5 | +5.3SD (BA) | NA | -1 | NA | -2 | 97 | -1.9* | NA | NA | 18.1 | NA | NA | [16] |
| c.301C>T(p.Gln101Ter) | F | 11.8 | -0.86SD  (BA) | NA | −1.3 | NA | −2.5 | 129.7 | −2.7* | NA | NA | 7.06 | NA | NA | [16] |
| c.7041delG（p.Cys2348ValfsTer8） | F | 13.9 | -0.6SD  (BA) | NA | −1.6 | NA | −1.5 | 140.5 | −2.9* | NA | NA | 13.80 | NA | NA | [16] |
| c.2099G>A(p.Trp700Ter) | F | 14.2 | +0.72SD  (BA) | NA | −0.2 | NA | −1 | 142.0 | −2.8* | NA | NA | 9.74 | NA | NA | [16] |
| c.2099G>A(p.Trp700Ter) | M | 10.8 | +1.35SD  (BA) | NA | −0.6 | NA | −1.5 | 130.8 | −1.8* | NA | NA | NA | 194 | 4.55 | [16] |
| c.410418delinsTGGA  (p.His137LeufsTer31) | M | 5 | +1.07SD  (BA) | NA | +0.2 | NA | −1 | 102.6 | −1.6* | NA | NA | 5.96 | 82 | 3.26 | [16] |
| c.560dupA | M | 10.8 | 0.7 | 2.8 | NA | 47 | NA | 120.7 | −3.47 | 24 | 16.44 | NA | NA | NA | [17] |
| c.1117_1120delCAGA | M | 15 | -2.5 | NA | NA | NA | NA | 133.4 | -5.51 | 29 | 16.3 | 5.94 | 139 | NA | [17] |
| c.1467C>G | M | 8.4 | 0.6 | 3.2 | NA | 49 | NA | 113.6 | -3.47 | NA | NA | NA | NA | NA | [17] |
| c.1861A>T | F | 6.5 | 4.5 | 2.1 | NA | 48 | NA | 102.5 | -3.67 | 17.6 | 16.75 | 9.34 | 191 | NA | [17] |
| c.1880_1883dupTGGC | M | 4 | 2 | NA | NA | NA | NA | 92 | -2.95 | 12.9 | 15.24 | 2.92 | 52.6 | NA | [17] |
| c.2173delG | M | 10 | 1.5 | 3.4 | NA | NA | NA | 118.5 | -3.5 | 23 | 16.4 | 23.23 | 143.3 | NA | [17] |
| c.5579delC | F | 9.4 | 1.3 | 3.95 | NA | 50 | NA | 119.5 | -2.91 | 22.2 | 15.55 | 5.91 | 149 | NA | [17] |
| c.6861delC | F | 9.3 | -2.8 | NA | NA | NA | NA | 115.2 | -3.31 | 19.5 | 14.7 | 7.2 | 282 | NA | [17] |
| c.4390delG(p.Val1464Ter) | F | 10.8 | 0.2 | 3.3 | -0.05 | 46 | -2.12 | 125.8 | -2.5 | 28.9 | 18.26 | 25.6 | 275 | NA | [26] |
| c.4390delG(p.Val1464Ter) | F | 7.6 | 0.0 | 3.1 | -0.85 | 46 | -2.34 | 109.6 | -2.4 | 18.6 | 18.25 | 27.9 | 132 | NA | [26] |
| c.1817C>A(p.A606D) | M | 2.8 | 0.2 | 3.4 | NA | 50 | NA | 87.4 | -2.02 | 13 | 17.02 | NA | NA | NA | [19] |
| c.1429+1G>T | F | 5.8 | 0.0 | 3.3 | NA | 50 | NA | 106.5 | -2 | 16.5 | 14.55 | 13.067 | 203 | NA | [19] |
| c.1429+1G>T | F | 10.8 | 0.7~1.2 | NA | NA | 50 | NA | 142.4 | -0.47 | 41 | 20.21 | NA | NA | NA | [19] |
| c.538G>A | M | 4.5 | 1.0 | 3.3 | NA | 50 | NA | 99.6 | -1.98 | 18 | 18.14 | 11.414 | 131 | NA | [19] |
| c.70+1G>A | M | 14.3 | -2.8 | 2.9 | NA | 50 | NA | 132.2 | -4.9 | 45 | 25.75 | 7.332 | 167 | NA | [19] |
| c.6665G>A | F | 8.2 | 0.6 | 2.6 | NA | NA | NA | 116.5 | -3.5 | 23 | 16.95 | 12.631 | 212 | NA | [19] |
| c.5058_5059delCA | M | 12.9 | 0.1 | 3.35 | NA | 50 | NA | 137.9 | -4.2 | 46.3 | 24.35 | 6.219 | 530 | NA | [19] |
| c.492C>A | F | 14 | 2.0 | NA | NA | NA | NA | 132.8 | -2.77 | 45.5 | 25.80 | NA | NA | NA | [19] |
| c.1032C>G | M | 11.2 | -0.2 | NA | NA | NA | NA | 126.9 | -2.9 | 33 | 20.49 | ＞10 | NA | NA | [19] |
| c.2398delinsA | F | 1.3 | NA | 4.45 | NA | 51 | NA | 71.3 | -2.88 | 8 | 15.74 | NA | NA | NA | [19] |
| c.3587delC | M | 3.1 | 0.9 | 3.35 | NA | 50 | NA | 87.2 | -2.7 | 14.2 | 18.67 | NA | 72.5 | NA | [19] |
| c.2173G>T | M | 10.1 | 0.4 | 3.1 | NA | NA | NA | 122.7 | -2.91 | 25.5 | 16.94 | ＞34.8 | 365 | NA | [19] |
| c.7198G>T | F | 3 | 1.2 | 3.38 | NA | 47 | NA | 87.3 | -2.24 | 12.3 | 16.14 | NA | 110 | NA | [19] |
| c.7465C>G | M | 7.3 | 0.2 | 3.1 | NA | 50 | NA | 114.8 | -2.1 | 26 | 19.73 | 8.46 | 135 | NA | [19] |
| Del exon12 | F | 1.5 | -1.0 | 2.5 | NA | NA | NA | 70.5 | -3.7 | 7.7 | 15.49 | NA | 46.6 | NA | [19] |
| c.1551C>G(p.Tyr517Ter) | M | 10.0 | 1.0 | NA | NA | NA | NA | NA | -3.00 | NA | NA | 8.6 | NA | NA | [27] |
| c.1551C>G(p.Tyr517Ter) | F | 7.2 | -0.2 | NA | NA | NA | NA | NA | -2.73 | NA | NA | 8.4 | NA | NA | [27] |
| c.217delA(p.Ile73SerfsTer12) | F | 7.0 | 0.4 | NA | NA | NA | NA | NA | -2.45 | NA | NA | 6.6 | NA | NA | [27] |
| c.1051+2T>A | M | 3 | -0.4 | 3.2 | NA | 50 | NA | 84 | −3.46 | NA | NA | NA | 37 | 2.81 | [28] |
| c.313T>C(p.S105P) | M | 3.1 | 0.5 | 3.3 | NA | 48 | NA | 83.2 | −3.65 | NA | NA | NA | 56 | 3.55 | [28] |
| c.2660C>G(p.S887X） | M | 2.9 | -0.9 | 2.44 | NA | 48 | NA | 85.5 | −3.05 | NA | NA | NA | 60 | 2.51 | [28] |
| c.2153C>A(p.T718K) | M | 7.3 | -0.9 | 3.1 | NA | 49 | NA | 103.1 | −3.22 | NA | NA | 7.39 | 151 | 4.54 | [28] |
| c.7243delG(p.D2415Tfs*4) | M | 9.4 | 0.6 | 3.3 | NA | 50 | NA | 125 | −2.18 | NA | NA | 2.33 | 205 | 5.28 | [28] |
| c.2911G>T(p.G971X) | M | 3.4 | 0.4 | 2.97 | NA | 49 | NA | 89 | −3.08 | NA | NA | NA | 91 | 3.44 | [28] |
| c.6_13delCACTTTAC  (p.Thr3Leufs*21) | M | 7.5 | -1.5~-0.5 | 4.1 | NA | NA | NA | 104.8 | -4.3 | NA | NA | 12.2 | NA | NA | [18] |
| c.661delT(p.Tyr221Metfs*10) | M | 12 | -0.5~0.5 | 2.9 | NA | 45 | −3.0 | 130 | -2.96 | 43.7 | 25.85 | 5.96 | NA | NA | [18] |
| c.1117_1120delCAGA(p.Thr374*) | M | 13.3 | -0.3~0.7 | NA | NA | NA | NA | 133.4 | -3.54 | 29 | 16.3 | 5.94 | NA | NA | [18] |
| c.845C>T(p.Thr282Ile) | F | 7.5 | 0.1 | 2.5 | NA | 50 | +0.18 | 105.8 | -3.8 | 16.5 | 14.74 | 23 | NA | NA | [20] |
| c.902G>A(p.Trp301*) | M | 6.3 | 0.2 | 3 | NA | 50 | -0.22 | 106 | -2.9 | 19 | 16.91 | 5.53 | NA | NA | [20] |
| c.6176del(p.Pro2059Leufs*2) | M | 6.6 | 0.9 | 2.7 | NA | 48 | -1.33 | 104.5 | -3.3 | 17.5 | 16.03 | 10.20 | NA | NA | [20] |
| c.7293del(p.Cys2432Alafs*139) | F | 8 | NA | NA | NA | NA | NA | 111 | -3.2 | NA | NA | NA | NA | NA | [20] |
| c.2023C>T(p.Arg675*) | M | 5.9 | 1.6 | 2.8 | NA | 48 | -1.33 | 100.4 | -3.8 | 15 | 14.88 | 19.9 | NA | NA | [20] |
| c.7230del（p.Phe2410Leufs*9） | M | 4 | 1.5 | NA | NA | NA | NA | 94.8 | -2.02 | 14.6 | 16.25 | NA | NA | NA | [21] |
| M c.130G>A p.(Gly44Arg) | M | 6.1 | 0.8 | NA | NA | NA | NA | NA | -2.5 | NA | NA | NA | NA | NA | [49] |
| Mc.130G>A p.(Gly44Arg) | M | 10.0 | 0.6 | NA | NA | NA | NA | NA | -2.3 | NA | NA | NA | NA | NA | [49] |
| c.454+1G>A p.? | F | 4.2 | 1.9 | NA | NA | NA | NA | NA | -3.0 | NA | NA | NA | NA | NA | [49] |
| c.706C>T p.(Arg236*) | F | 7.8 | 1.6 | NA | NA | NA | NA | NA | −2.2 | NA | NA | NA | NA | NA | [49] |
| c.706C>T p.(Arg236*) | M | 8.7 | 0.0 | NA | NA | NA | NA | NA | −2.3 | NA | NA | NA | NA | NA | [49] |
| c.706C>T p.(Arg236*) | M | 9.2 | 0.0 | NA | NA | NA | NA | NA | -3.1 | NA | NA | NA | NA | NA | [49] |
| c.775_784delins17  p.(Thr259 fs) | M | 14.9 | −5.0 | NA | NA | NA | NA | NA | −2.8 | NA | NA | NA | NA | NA | [49] |
| M c.1608C>A p.(Tyr536*) | M | 5.0 | 0.9 | NA | NA | NA | NA | NA | -3.2 | NA | NA | NA | NA | NA | [49] |
| M c.1608C>A p.(Tyr536*) | F | 5.0 | 0.0 | NA | NA | NA | NA | NA | -3.7 | NA | NA | NA | NA | NA | [49] |
| M c.1608C>A p.(Tyr536*) | M | 11.9 | 0.6 | NA | NA | NA | NA | NA | -2.4 | NA | NA | NA | NA | NA | [49] |
| c.2735_2736delins31  p.(Leu912fs) | F | 4.2 | -2.1 | NA | NA | NA | NA | NA | -4.8 | NA | NA | NA | NA | NA | [49] |
| c.2735_2736delins31  p.(Leu912fs) | F | 11.9 | 1.4 | NA | NA | NA | NA | NA | -2.8 | NA | NA | NA | NA | NA | [49] |
| c.4700del  p.(Ser1567Metfs*10) | F | 6.0 | 0.0 | NA | NA | NA | NA | NA | -3.7 | NA | NA | NA | NA | NA | [49] |
| c.4762_4765del  p.(Gly1588Cysfs*26) | M | 12.5 | 1.0 | NA | NA | NA | NA | NA | -2.8 | NA | NA | NA | NA | NA | [49] |
| c.6673C>T p.(Gln2225*) | M | 10.9 | 0.3 | NA | NA | NA | NA | NA | -2.2 | NA | NA | NA | NA | NA | [49] |
| c.6673C>T p.(Gln2225*) | M | 12.6 | 0.2 | NA | NA | NA | NA | NA | -2.0 | NA | NA | NA | NA | NA | [49] |
| c.7096C>T p.(Gln2366*) | M | 4.3 | 1.0 | NA | NA | NA | NA | NA | -3.5 | NA | NA | NA | NA | NA | [49] |
| c.7204C>T p.(Gln2402*) | M | 12 | 0.5 | NA | NA | NA | NA | NA | -2.8 | NA | NA | NA | NA | NA | [49] |
| c.7255G>Cp.(Asp2419His) | F | 11.6 | 0.5 | NA | NA | NA | NA | NA | -1.6 | NA | NA | NA | NA | NA | [49] |
| c.7255G>Cp.(Asp2419His) | M | 14.3 | -1.0 | NA | NA | NA | NA | NA | -2.1 | NA | NA | NA | NA | NA | [49] |
| c.7602G>A p.(Trp2534*) | F | 5.9 | 0.0 | NA | NA | NA | NA | NA | -2.5 | NA | NA | NA | NA | NA | [49] |
| c.7602G>A p.(Trp2534*) | M | 14.3 | -0.5 | NA | NA | NA | NA | NA | -2.4 | NA | NA | NA | NA | NA | [49] |
| c.7602G>A p.(Trp2534*) | M | 15.7 | -2.5 | NA | NA | NA | NA | NA | -3.1 | NA | NA | NA | NA | NA | [49] |

*BA* bone age,*GH* growth hormone,*SD* standard deviation,*BMI* body mass index,*IGF1* insulin-like growth factor 1,*IGFBP3* insulin-like growth factor binding factor 3,*NA* not available，**indicates SGA,*indicates Sibling(first-degree relative)short but parent not short

**Supplementary Tables S2 Data on treatment with rhGH in patients with ACAN gene variant**

| ACAN Variant | Sex | GH starting age(y) | Height SDS at Start GH | Height SDS at end GH | ΔSDS | AH SDS | 1st year growthΔSDS | ΔSDS/y | Treatment | Reference |
| --- | --- | --- | --- | --- | --- | --- | --- | --- | --- | --- |
| c.4852C>T（p.Gln 1618*） | M | 11.8 | -4.0 | -3.8 | +0.2 | NA | +0.2 | +0.2^b^ | GH(0.29 mg/kg/w,11m) | [22] |
| c.7465 T>C(p.Gln2364Pro) | F | 5.6 | -2.09 | -1.07 | +1.02 | NA | +0.33 | +0.26 | GH(40-50μg/kg/d,47 m)  +GnRHa(30 m) | [24] |
| c.7465 T>C(p.Gln2364Pro) | M | 6.4 | -3.74 | -3.75 | -0.01 | NA | +0.23 | -0.00 | GH(60μg/kg/d,92 m) | [24] |
| t(10;15)(q22.3;q26.1) | F | 12.0 | -2.80 | -2.82 | -0.02 | NA | -0.03 | -0.00 | GH(0.025-0.037mg/kg/d,41m) | [23] |
| t(10;15)(q22.3;q26.1) | F | 9.6 | -2.34*^a^* | -2.26*^a^* | +0.08 | -2.37 | +0.16*^a^* | +0.02 | GH(0.035-0.05 mg/Kg/d,44m)  +GnRHa(39 m) | [23] |
| c.5026_5027 del  （p.Ser 1676 Ter） | M | 4.1 | -3.6 | -1.7 | +1.9 | NA | +1.8 | +1.2 | GH(50μg/kg/d,18 m) | [29] |
| c.5026_5027 del  (p.Ser 1676 Ter) | F | 5.5 | -3.4 | -3.1 | +0.3 | NA | NA | +1.2^b^ | GH(50μg/kg/d,3 m) | [29] |
| c.1504C>T(p.R 502 C) | M | 3.0 | -3.9 | -2.86 | +1.04 | NA | +1.3 | +0.57 | GH(33μg/kg/d,22 m) | [29] |
| c.1817C>A(p.Ala606Asp) | M | 9.4 | -2.9 | -1.9 | +1.0 | NA | NA | +0.4 | GH(NA,30 m) | [25] |
| c.2266G>C(p.Gly756Arg) | M | 3.9 | -2.9 | -2.2 | +0.7 | NA | NA | +0.8^b^ | GH(NA,11 m) | [25] |
| c.7469G>A(p.Cys2490Tyr) | M | 15.1 | -4.5 | -4.4 | +0.1 | NA | NA | +0.6^b^ | GH(NA,2 m)+GnRHa(2 m) | [25] |
| c.711051del（p.His25Thr350del） | F | 3.3 | -2.5 | -1.9 | +0.6 | NA | NA | +0.5 | GH(34.8µg/kg/d,14 m) | [16] |
| c.301C>T(p.Gln101Ter) | F | 7.9 | -4.3 | −2.7 | +1.6 | NA | NA | +0.38 | GH(34.8μg/kg/d,51 m) | [16] |
| c.7041delG（p.Cys2348ValfsTer8） | F | 11.7 | -2.5 | −2.9 | +0.4 | NA | NA | +0.18 | GH(34.8μg/kg/d,27 m)  +GnRHa(11.25 mg/Q3m) | [16] |
| c.2099G>A(p.Trp700Ter) | F | 11.0 | -2.5 | −2.8 | -0.3 | NA | NA | +0.09 | GH(34.8μg/kg/d,38 m)  +GnRHa(11.25 mg/Q3m) | [16] |
| c.2099G>A(p.Trp700Ter) | M | 10.6 | -2.0 | −1.8 | +0.2 | NA | NA | +0.8^b^ | GH(34.8μg/kg/d,3 m)  +GnRHa(11.25 mg/Q3m) | [16] |
| c.5579delC(p.Gly1861fs) | F | 9.5 | -2.9 | -2.3 | +0.6 | NA | NA | +0.4 | GH(48μg/kg/d,19 m) | [17] |
| c.1817C>A(p.A606D) | F | 2.9 | -2.1 | -1.4 | +0.7 | NA | NA | +1.4^b^ | GH(40μg/kg/d,6 m) | [19] |
| c.1429+1G>T(splicing) | F | 6.0 | -2 | -1.4 | +0.6 | NA | +0.6 | +0.6 | GH(53μg/kg/d,12 m) | [19] |
| c.538G>A(p.A180T) | F | 4.5 | -2 | -0.9 | +1.1 | NA | NA | +0.9 | GH(53μg/kg/d,14 m) | [19] |
| c.70+1G>A(splicing) | M | 14.3 | -4.9 | -5 | -0.1 | NA | NA | -0.8^b^ | GH(43μg/kg/d,1.5 m) | [19] |
| c.5058_5059delCA  (p.I1686Mfs*13) | M | 12.8 | -2.7 | -2.6 | +0.1 | NA | NA | +0.2^b^ | GH(53μg/kg/d,5 m)  +letrozole(2.5 mg qd) | [19] |
| c.1551C>G(p.Tyr517Ter) | M | 10 | -3.00 | -2.78(1) | +0.22 | NA | +0.22 | +0.22 | GH(28.6μg/kg/d，12m) | [27] |
| c.1551C>G(p.Tyr517Ter) | F | 7.2 | -2.73 | -2.48(1) | +0.25 | NA | +0.25 | +0.25 | GH(32.9μg/kg/d，12m) | [27] |
| c.217delA(p.Ile73SerfsTer12) | F | 7 | -2.45 | -2.13(1) | +0.32 | NA | +0.32 | +0.32 | GH(32.9μg/kg/d，12m) | [27] |
| c.1051+2T>A | M | 3 | −3.46 | −3.13 | +0.33 | NA | NA | +0.33^b^ | GH(0.139-0.171IU/kg/d,6m) | [28] |
| c.313T>C(p.S105P) | M | 3.1 | −3.65 | −2.68 | +0.97 | NA | NA | +0.97^b^ | GH(0.139-0.171IU/kg/d,8m) | [28] |
| c.2660C>G(p.S887X） | M | 2.9 | −3.05 | −1.35 | +1.7 | NA | NA | +1.7 | GH(0.139-0.171IU/kg/d,63m) | [28] |
| c.2153C>A(p.T718K) | M | 7.3 | −3.22 | −1.37 | +1.85 | NA | NA | +1.85 | GH(0.139-0.171IU/kg/d,20m) | [28] |
| c.7243delG(p.D2415Tfs*4) | M | 9.4 | −2.18 | −0.44 | +1.74 | NA | NA | +1.74 | GH(0.139-0.171IU/kg/d,33m) | [28] |
| c.2911G>T(p.G971X) | M | 3.4 | −3.08 | −2.46 | +0.62 | NA | NA | +0.62^b^ | GH(0.139-0.171IU/kg/d,3m) | [28] |
| c.845C>T(p.Thr282Ile) | F | 8.2 | -3.8 | -3.2 | +0.6 | NA | NA | +0.54 | GH(33-39.6μg/kg/d,,13m) | [20] |
| c.902G>A(p.Trp301*) | M | 6.3 | -2.9 | -2.4 | +0.5 | NA | NA | +0.71^b^ | GH(33μg/kg/d,8.4 m) | [20] |
| c.6176del  (p.Pro2059Leufs*2) | M | 6.6 | -3.3** | -3.0 | +0.3 | NA | NA | +0.45^b^ | GH(56.1–62.7μg/kg/d,8m) | [20] |
|  |  | 11.8* | -2.5** | -2.4 | +0.1 | NA | NA | +0.33^b^ | GH(49.5μg/kg/d,4 m) |  |
| c.2023C>T(p.Arg675*) | M | 6 | -3.8** | -3.0 | +0.8 | NA | NA | +0.53 | GH(56.1-62.7,μg/kg/d,18m) | [20] |
| c.7230del（p.Phe2410Leufs*9） | M | 4.6 | -2.6 | -1.8 | +0.8 | NA | +0.8 | +0.8 | GH(50μg/kg/d,13 m) | [21] |
| M c.130G>A p.(Gly44Arg) | M | 6.1 | -2.5 | NA | NA | NA | NA | NA | GH(NA,NA) | [49] |
| Mc.130G>A p.(Gly44Arg) | M | 10.0 | -2.3 | NA | NA | NA | NA | NA | GH(NA,NA) | [49] |
| c.454+1G>A p.? | F | 4.2 | -3.0 | NA | NA | NA | NA | NA | GH(NA,NA) | [49] |
| c.706C>T p.(Arg236*) | F | 7.8 | −2.2 | NA | NA | NA | NA | NA | GH(NA,NA) | [49] |
| c.706C>T p.(Arg236*) | M | 8.7 | −2.3 | NA | NA | NA | NA | NA | GH(NA,NA) | [49] |
| c.706C>T p.(Arg236*) | M | 9.2 | -3.1 | NA | NA | NA | NA | NA | GH(NA,NA) | [49] |
| c.775_784delins17  p.(Thr259 fs) | M | 14.9 | −2.8 | NA | NA | NA | NA | NA | GH(NA,NA) | [49] |
| M c.1608C>A p.(Tyr536*) | M | 5.0 | -3.2 | NA | NA | NA | NA | NA | GH(NA,NA) | [49] |
| M c.1608C>A p.(Tyr536*) | F | 5.0 | -3.7 | -4.0 | -0.3 | NA | NA | NA | GH(NA,NA) | [49] |
| M c.1608C>A p.(Tyr536*) | M | 11.9 | -2.4 | -1.3 | +1.1 | NA | NA | NA | GH(67μg/kg/d,NA) | [49] |
| c.2735_2736delins31  p.(Leu912fs) | F | 4.2 | -4.8 | NA | NA | NA | NA | NA | GH(NA,NA) | [49] |
| c.2735_2736delins31  p.(Leu912fs) | F | 11.9 | -2.8 | NA | NA | NA | NA | NA | GH(NA,NA) | [49] |
| c.4700del  p.(Ser1567Metfs*10) | F | 6.0 | -3.7 | NA | NA | NA | NA | NA | GH(NA,NA) | [49] |
| c.4762_4765del  p.(Gly1588Cysfs*26) | M | 12.5 | -2.8 | -2.6 | +0.2 | NA | NA | NA | GH(NA,NA) | [49] |
| c.6673C>T p.(Gln2225*) | M | 10.9 | -2.2 | NA | NA | NA | NA | NA | GH(NA,NA) | [49] |
| c.6673C>T p.(Gln2225*) | M | 12.6 | -2.0 | -1.6 | +0.4 | NA | NA | NA | GH(NA,NA) | [49] |
| c.7096C>T p.(Gln2366*) | M | 4.3 | -3.5 | NA | NA | NA | NA | NA | GH(NA,NA) | [49] |
| c.7204C>T p.(Gln2402*) | M | 12 | -2.8 | -2.4 | +0.4 | NA | NA | NA | GH(NA,NA) | [49] |
| c.7255G>Cp.(Asp2419His) | F | 11.6 | -1.6 | -2.3 | -0.7 | NA | NA | NA | GH(NA,NA) | [49] |
| c.7255G>Cp.(Asp2419His) | M | 14.3 | -2.1 | NA | NA | NA | NA | NA | GH(NA,NA) | [49] |
| c.7602G>A p.(Trp2534*) | F | 5.9 | -2.5 | NA | NA | NA | NA | NA | GH(NA,NA) | [49] |
| c.7602G>A p.(Trp2534*) | M | 14.3 | -2.4 | -1.9 | +0.5 | NA | NA | NA | GH(NA,NA) | [49] |
| c.7602G>A p.(Trp2534*) | M | 15.7 | -3.1 | NA | NA | NA | NA | NA | GH(NA,NA) | [49] |

*y* years,*m* month,*NA* not available,*GH* growth hormone,*SDS* standard deviation score,*GnRHa* gonadotropin releasing hormone agonist,*AH* adult height,

*a* indicates obtained from growth charts*,b* indicates less than 1 year of treatment*,***indicates SGA,*ΔSDS* indicates Height SDS at end GH-Height SDS at Start GH

**Supplementary Tables S3 Data on treatment with rhGH in patients with variant in the SHOX gene**

| SHOX Variant | Sex | GH starting age(y) | Height SDS at Start GH | Height SDS at end GH | ΔSDS | ΔSDS/y | Treatment | Reference |
| --- | --- | --- | --- | --- | --- | --- | --- | --- |
| c.577G>A(p.Ala193Thr) | F | 8 | -4.31 | -2.37 | +1.94 | +1.39 | GH(0.15IU/kg/d,17m) | [31] |
| Deletion | M | 4 | -3.6** | -2.66 | +0.94 | +0.19 | GH(0.04mg/d,60m) | [54] |
| Deletion | M | 7.5 | -3.2 | -2.68 | +0.52 | +0.52 | GH(NA,12m) | [54] |
| c.1044G>T  (p.Arg118Met) | M | 6 | -2.05 | -1.65 | +0.4 | +0.4 | GH(0.03 mg/kg/d,12m) | [53] |
| enhancer duplication | M | 3.8 | -3.4 | -2.7 | +0.7 | +0.7 | GHNA,12m) | [52] |

*y* years,*m* month,*GH* growth hormone,*SDS* standard deviation score,*ΔSDS* indicates Height SDS at end GH-Height SDS at Start GH,

*NA* not available,****indicates SGA

**Supplementary Tables S4 Data on patients with NPR2 gene variant treated with rhGH**

| NPR2 Variant | Sex | GH starting age(y) | Height SDS at Start GH | Height SDS at end GH | ΔSDS | 1st year growthΔSDS | ΔSDS/year | Treatment | Reference |
| --- | --- | --- | --- | --- | --- | --- | --- | --- | --- |
| c.109_119del  p.(Ser37Glyfs*37) | M | 4.3 | -2.7 | NA | NA | NA | NA | GH(33~40μg/kg/d,NA) | [58] |
| c.109_119del  p.(Ser37Glyfs*37) | M | 5.3 | -4.4 | NA | NA | NA | NA | GH(33~40μg/kg/d,NA) | [58] |
| c.109_119del  p.(Ser37Glyfs*37) | M | 12.8 | -2.7 | NA | NA | NA | NA | GH(33~40μg/kg/d,NA) | [58] |
| c.224G>T  p.(Cys75Phe) | F | 11.4 | -2.5 | NA | NA | NA | NA | GH(33~40μg/kg/d,NA)+GnRHa | [58] |
| c.239C>A  p.(Ala80Glu) | F | 11.9 | -2.3 | NA | NA | NA | NA | GH(33~40μg/kg/d,NA)+GnRHa | [58] |
| c.239C>A  p.(Ala80Glu) | F | 11.7 | -2.6 | NA | NA | NA | NA | GH(33~40μg/kg/d,NA) | [58] |
| c.298G>C  p.(Gly100Arg) | M | 18 | -2.4 | NA | NA | NA | NA | GH(33~40μg/kg/d,NA)+AI | [58] |
| c.298G>C  p.(Gly100Arg) | M | 15.2 | -2.1 | NA | NA | NA | NA | GH(33~40μg/kg/d,NA)+AI | [58] |
| c.1225G>A  p.(Ala409Thr) | M | 6.2 | -2.9 | NA | NA | NA | NA | GH(33~40μg/kg/d,NA) | [58] |
| c.1566_1567del  p.(Ser523Leufs*32) | M | 6.3 | -2.9 | NA | NA | NA | NA | GH(33~40μg/kg/d,NA) | [58] |
| c.1730delinsGTTTTTTCTG  p.(AsndelinsSerPhePheCys) | M | 10.5 | -3.2 | NA | NA | NA | NA | GH(33~40μg/kg/d,NA) | [58] |
| c.1887+2T>A | M | 4.6 | -3.2 | NA | NA | NA | NA | GH(33~40μg/kg/d,NA) | [58] |
| c.1954G>A p.(Val652Met) | F | 11.9 | -3.8 | NA | NA | NA | NA | GH(33~40μg/kg/d,NA) | [58] |
| c.2453_2456del  p.(Glu818Alafs*28) | M | 8.3 | -4.7 | NA | NA | NA | NA | GH(33~40μg/kg/d,NA) | [58] |
| c.2455C>T p.(Arg819Cys) | F | 13.6 | -3.0 | NA | NA | NA | NA | GH(33~40μg/kg/d,NA) | [58] |
| c.2678T>C p.(Phe893Ser) | M | 11.7 | -3.4 | NA | NA | NA | NA | GH(33~40μg/kg/d,NA) | [58] |
| c.2720C>T p.(Thr907Met) | F | 8.8 | -3.7 | NA | NA | NA | NA | GH(33~40μg/kg/d,NA) | [58] |
| c.2643 G>A(p.Gln811Gln) | M | 7.5 | -3.11 | -2.77 | +0.36 | NA | +0.72 | GH(0.050 mg/kg/d,6 m) | [35] |
| c.2298_2299delGCinsTT  (p.Glu766_Arg767delinsAspStop) | F | 9.0 | -1.88 | -0.66 | +1.22 | +1.07 | +0.83 | GH(0.043 mg/kg/d,18 m)+GnRHa(12 m) | [35] |
| c.613C>T(p.Arg205*) | M | 7 | -2.9 | -1.5 | +1.40 | +0.65 | +0.70 | GH(33μg/kg/d,24 m) | [36] |
| c.1673T>C(p.Ile558Thr) | F | 3 | -3.7 | -2.1(5)* | +1.80 | +0.79 | +0.36 | GH(33μg/kg/d,108 m) | [36] |
| c.1673T>C(p.Ile558Thr) | F | 10 | -3.0 | -1.8 | +1.20 | +0.33 | +0.24 | GH(33μg/kg/d,60 m) | [36] |
| c.1808G>C(p.Ser603Thr) | M | 4 | -4.0 | -2.2 | +1.80 | +0.65 | +0.36 | GH(33μg/kg/d,60 m) | [36] |
| c.1670G>A(p.Arg557His) | F | 3 | -3.8 | -2.2(5)* | +1.60 | +0.65 | +0.32 | GH(33μg/kg/d,72 m) | [36] |
| c.2761C>T(p.Arg921Ter) | F | 7.8 | -2.5 | 0.0(2)* | +2.50 | +1.40 | +1.25 | GH(0.3-0.34 mg/kg/w,NA) | [57] |
| c.2905G>C(p.Val969Leu) | M | 5.5 | -3.2 | -1.8 | +1.4 | NA | +0.42 | GH(50μg/kg/day,40m) | [56] |
| c.1167G>T(p.E389D) | F | 6 | -3.84 | -3.14 | +0.70 | NA | +0.06 | GH(NA，144m) | [47] |
| c.1481T>G(p.Ile494Ser) | F | 6 | -3.57 | -1.79 | +1.78 | NA | +0.36 | GH(NA，60m) | [47] |
| c.226T＞C(p.Ser76Pro) | M | 13^a^ | -2.1 | -2.1(2)* | 0 | NA | 0 | GH(50μg/kg/d,NA)+letrozole(2.5mg/d) | [55] |
| c.788G＞C(p.Arg263Pro) | M | 14^a^ | -2.8 | -2.5 | +0.3 | NA | +0.09 | GH(33μg/kg/d,40m)+GnRHa(18 m) | [55] |
| c.2455C T(p.Arg819Cys) | M | 13 | -2.8 | -2.4(4)* | +0.4 | NA | +0.1 | GH(50μg/kg/d,48m)+GnRHa(32 m) | [55] |
| c.1162 C>T(p.R388) | M | 10.3 | -2.71 | -3.08 | -0.47 | NA | NA | GH(NA,5m);GH(NA,6m)^b^ | [59] |

*y* years,*m* month,*GH* growth hormone,*SDS* standard deviation score,*ΔSDS* indicates Height SDS at end GH-Height SDS at Start GH,*NA* not available,*AI* aromatase inhibitor

*indicates last recorded height SDS but still subsequently treated with GH,*a* indicates Presence of the variant in heterozygous state,*b* indicates With an interval of 2 years
